# Academic activism: choosing the right time and the right place

**DOI:** 10.1038/s41390-024-03628-z

**Published:** 2024-10-15

**Authors:** Bryan Stevenson, Michael R. DeBaun

**Affiliations:** 1 Equal Justice Initiative, Montgomery, AL USA; 2 Vanderbilt-Meharry, Center of Excellence in Sickle Cell Disease, Vanderbilt University School of Medicine, Nashville, TN USA

As APS president 2023-24, I have focused much of my energy on the APS issue of the year: Increasing Access to Quality Healthcare for Children and Adolescents Who are Incarcerated (2023–2024). The APS Presidential Plenary highlighted our effort to activate child healthcare leaders to engage in a multi-disciplinary strategy for improving the healthcare of youth in custody. Also, during the Presidential Plenary, Bryan Stevenson JD, the founder and director of the Equal Justice Initiative (Home (eji.org), provided a powerful point about our carceral system and the need for just mercy in supporting our youth held in custody. Other tangible APS actions related to the year’s issue was the summer 2023 council meeting in Montgomery, Alabama, where we visited the Legacy Museum from Enslavement to Mass Incarceration and the National Memorial for Peace and Justice. The visit set the tone and provided the background and context for the APS council to embrace the APS issue of the year.

Other tangible APS outcomes for the issue of the year have been multiple commentaries published in our official journal, *Pediatric Research*, emphasizing several of the biggest challenges for this invisible population: (1) APS issue of the year Increasing access to quality healthcare for children who are incarcerated: American Pediatric Society issue of the year (2023–2024),^1^ (2) Time-sensitive healthcare guidelines for youth with chronic diseases in custody: gaps in care,^2^ (3) Enhancing medical education: youth in custody,^3^ (4) Medicaid policy changes are set to allow coverage for some health services for youth in custody: what should pediatricians know?,^4^ (5) Increasing access to sexual and reproductive health care for youth in custody.^5^ With the help of the APS Advocacy Policy Committee, we have elected to reach out to the medical professional societies that write evidence-based guidelines for medical care for children and adolescents and encourage these societies to at least mention the invisible population of youth health in custody and provide reasonable minimum health care standards for this population.

The American Society of Hematology has been the first to embrace this new quality care objective. In May 2024, we met in Washington DC, with 6 carceral experts and 3 sickle cell disease experts to address this gap in the American Society of Hematology’s evidence-based guidelines for children and adults with SCD held in custody. The small group has been tasked to move forward with a commissioned commentary to adapt the ASH time-sensitive SCD guidelines for those in custody. We have completed a commissioned commentary to be submitted to Blood Advances describing expected standard care for children an adults with sickle cell disease in custody.

The APS’s Advocacy Committee of APS will continue to identify other gaps in evidence-based guidelines for youth in custody and will continue to solicit changes in these guidelines to at least acknowledge our youth in custody. A special thanks to Elizabeth Barnert, MD, newly elected APS member, who has been my tutor and partner in educating me about the challenges of this special population of children and working with me to advance APS’s issue of the year.

We introduced a pilot program, the APS President Book Club, which has been a resounding success. I am grateful to the moderators and the participants who have supported enlightening conversations regarding the selected books. The books were ‘a Lab of One’s Own — One Women’s Personal Journey Through Sexism in Science’ by Rita Colwell, PhD, and Sharon Bertsch McGrayne; ‘The Deepest Well’ by Nadine Burke Harris, MD; ‘Caste: The Origins of Our Discontents’ by Isabel Wilkerson; ‘Just Mercy: A Story of Justice and Redemption’ by Bryan Stevenson, JD; ‘The Sum of Us: What Racism Costs Everyone and How We Can Prosper Together’ by Heather McGhee, JD; and ‘GRIT: The Power of Passion and Perseverance’ by Angela Duckworth, PhD. I want to extend a special thanks to the moderators who ventured into the uncharted territory of a virtual APS Book Club – Lisa Satlin, Peggy Hostetter, Harold Lehmann, Lisa Robinson, Elizabeth Barnert, Katie Plax, and Joseph St. Geme, III. Through this initiative, we have learned about each other and explored ways to enhance our academic mission of advancing the medical care for our children.

APS leadership also teamed with SPR leadership to create the first-Gordon conference for our APS-SPR Journeys recipients at PAS, which had 62 attendees. The conference was a success, and I want to acknowledge the Burroughs Wellcome Foundation, which co-sponsored the event.

Also, for the first time at PAS, APS with SPR sponsored 50 medical students, residents, and physician-scientist trainees from the University of Toronto Medical School focused on improving child health. This urgent effort was our immediate response to the 2024 March match results in pediatrics, revealing the low number of medical school graduates electing a pediatric residency. Special recognition for this timely response goes to the APS Council, APS staff- Lisa Thompson, Michelle Brunoehler, and Shelly Job, immediate APS Past President Lisa Robinson, President-elect Cliff Bogue, and Secretary-Treasurer Catherine Gordon, who all supported a timely response to register and host the 50 trainees at PAS in less than 30 days!

On a more personal note, one of the year’s highlights was having dinner and coffee the following morning at Alan Jobe’s home, the 2024 Howland Awardee, in December of 2023. Rarely do you have an opportunity in your career to share a meal with a pioneer in the field who has meant so much to so many.

In summary, thank you for a great year as APS President. Although my time has ended, the memories will last a lifetime. Here is the interview with the APS president’s keynote speaker Bryan Stevenson, JD.

**Transcript of May 29, 2024, Conversation Between Michael DeBaun MD, MPH, MD, and Bryan Stevenson MPP/JD, LDD**

**Michael DeBaun MD, MPH**: Good morning, Bryan.

**Bryan Stevenson JD**: How are you?

**Michael DeBaun MD, MPH:** You know, I’m loving life, man. How are you doing?

**Bryan Stevenson JD**: I’m great. Thank you.

**Michael DeBaun MD, MPH:** Well, I appreciate your support. So, I have the idea here, the big picture is to have a dialogue so that we can introduce this concept of improving health care for children and adolescents who are held in custody to a predominantly child health academic audience.

So, the APS members, by definition, are individuals who have a national footprint in child health, covering the complete spectrum of academics. The leaders in our society have not explicitly focused on improving or advancing the medical care for this high risk invisible population, children and adolescents held in custody and newborn delivered in custody. What I’d like this interview to do is hear your voice on how we can partner moving forward in this space of improving health care for newborns, children, and adolescents in custody.

**Bryan Stevenson JD:** Perfect

**Michael DeBaun MD, MPH:** Okay. So, let me start off with the issue of the year: **Improving Quality Health Care for Children and Adolescents Held in Custody**.^1^ What would the ideal world look like for children who are held in custody and providing them quality health care upon entering a facility, or a setting, and then leaving that setting and re -entering outside of a confinement or custody? What would that look like to you, Bryan?

**Bryan Stevenson JD:** I think it would begin with a health assessment about how this child came to be in a custodial setting in the first place. We don’t ask the question, why are children in these spaces?

And I think our inattention to the why question means that our response to a lot of these children ends up being based on assumptions, old ideas, incomplete information, and they tend to be ineffective.

And when a patient comes to any physician in the free world, the first thing we ask is, okay, why are you here? What are the symptoms? What are the challenges? What are the problems that you are experiencing?

And sometimes they go to the wrong physician, and we have to say, oh, no, you need to see an oncologist about that, or you need to see an orthopedist about that kind of pain. And we don’t do that generally with children in custodial settings.

We say, okay, you’re in this facility, and we’re going to respond to this, and we’re going to respond to that, but we’re not going to make that health assessment. So, I think the first thing is that we would really take seriously the obligation to understand why this child is in a situation where they now find their liberty and freedom constrained, their life constrained, and, in many ways, permanently compromised by incarceration.

The assessment requires the same kind of extensive history information that we would get if we’re trying to figure out a complex problem for a patient in some other setting. Creating trust so that the patient speaks honestly about their experiences and their history, and then letting them know that we’re here to evaluate health. We’re not here to judge behavior or to punish. We’re here to evaluate, assess and most importantly help.

This would transform the capacity of healthcare providers to improve child health. And it’s just generally not done. It tends to be superficial, it tends to be reactive to some symptom that we are seeing on display in the custodial setting, when assessment should be the baseline for child health in custodial settings for every person.

What we’ll learn, and you and I have experienced this, is that a lot of people are in these settings because they are dealing with trauma. They’re dealing with histories of abuse and victimization, they’re dealing with mental health problems that have been undiagnosed, that they’ve just been judged based on their behaviors, they’re dealing with impulses and emotions that they don’t have the language to describe and define. And so, it gets presented again, as pathology and bad behavior and all of these other labels.

With improved assessment and diagnosis, we can better access the tools for helping people recover from trauma. We have tools for helping people recover from abuse. We have tools for treating mental illness. We have tools for managing and addressing all of the complex health issues that people present. But without that baseline assessment, I just think we’re not going to even begin to have the kind of impact that many of us would like to see.

**Michael DeBaun MD, MPH:** Yes, you’re advocating for physicians to do what they’re obligated to do when they get a patient for the first time walk into the office, right? You get to know the patient, you take a history, you perform a physical examination, you have your laboratory evaluation, then you have an assessment with a differential diagnosis and a plan that’s evidence -based. 

**Bryan Stevenson JD:** That’s exactly right. And we should see the placement of a child in a custodial setting to be the condition that mandates that kind of evaluation because children shouldn’t be in custody.

Children free from all these issues shouldn’t need incarceration. And so, it’s the unhealthy situation of children in custody that prompts the need for a health evaluation of just the kind you describe.

**Michael DeBaun MD, MPH:** All right, so let’s say that this is the ideal world. We provide the standard care for newborns, children and adolescents in custody. Now, what would be the ideal setup for entry after they have been taken out of their primary care provider’s care, and held in custody?

**Bryan Stevenson JD:** Well, I mean, obviously this will turn on the diagnoses and the needs, but I think for all of these, patients, these children, we’d want to have a health narrative about what they need. Again, when someone’s diagnosed with a cancer, there’s a health narrative about what people must get to survive and no one questions that.

You know, if the oncologist says, oh, this person needs eight weeks of radiation or whatever, everybody says, okay, we got it. That’s something we must do, again, with other kinds of physical injuries. We say, oh no, this person needs to be in a cast for this period. That’s not usually questioned. We will want to provide that kind of health narrative, that health prescription, if you will, for people coming out of custody. And we must make it a health narrative so that others don’t question it or doubt it or undermine it because it seems like something extravagant or unnecessary. I’d say that’s number one.

Number two, we must help children recover from all the socio-emotional challenges that incarceration or these detention experiences create. We can’t be indifferent to the fact that the very experience of being in a carceral setting can create collateral problems.

And we can minimize a lot of those problems by helping people understand what those problems are. So, for example, we represent a lot of people who went to jails and prisons at 13, 14, 15, before they had any free world experience living as an adult. But they come out at 30 or 35, they look like full grown adults, and people will judge them negatively when they don’t show skills at being able to open a bank account or be able to pay rent or to manage independent living.

They’ll think that they’re not trying, or they’re just not motivated or that they’re compromised, when in fact they haven’t been given the help that they need uniquely. And so, what we do is say, we’re going to have to help you now develop skills that you would have developed if you hadn’t been incarcerated.

And we spend a lot of time on these life skills that others might think of as gratuitous, but we see as essential because they’ve been denied the opportunity for that. Relationship skills are important. We should not underestimate the challenges that children in custody have in creating healthy relationships upon re-entry, because the modes of relationship that led them to be in custody in the first place were frequently unhealthy. Relationships within these institutions aren’t always healthy. So having someone talk with you about that and help you think differently about that, that’s another area where we spend a lot of time that we find that to be critical to the wellbeing and the success of people, particularly young people coming out. I mean, even without incarceration, young people have a lot of anxiety and stress about relationships. It’s such a powerful force in the life of an adolescent.

**Michael DeBaun MD, MPH:** You could not have made a stronger argument for the role of a general pediatrician, once a child or adolescent enters custody. Also, you make a very compelling argument for continuity of care. In fact, many pediatricians go into pediatrics, specifically general pediatrics, because they can follow their patients for 20 plus years, a unique component of our field.

**Bryan Stevenson JD:** That’s right. And I think that’s so important. And you’re right, doc. In some areas, we accept this without question. So, if someone breaks a bone and their release is going to come in the middle of the treatment, no one questions that you can’t stop treatment for that broken bone after two weeks when an eight-week treatment is needed. Same for oncology. And I think that continuity of care is well established in many areas. We just have to make that equally well established when it comes to continuity of care for children coming into these facilities and leaving.

**Michael DeBaun MD, MPH:** Perfect. I couldn’t agree with you more. Now, I want you to put on another hat.

You are wearing the hat of the chief marketing person advocating for best available care for children in custody. When you’re wearing this hat, your job is to convince the department chairs of pediatrics throughout the country, the division chiefs of adolescent medicine throughout the country, the heads of ambulatory pediatrics throughout the country.

These are leaders, many of whom are members of the American Pediatric Society. What is the narrative? What is the compelling argument? What’s in it for them to embrace this pediatric population that frankly has been ignored? How would you make the compelling case that providing medical care for this population is impactful?

**Bryan Stevenson JD:** I would begin with just the premise that most practitioners and chairs are doing this because they want to improve the health of the community.

I would argue that the health of the community must be measured not by what we do for privileged kids and talented kids and gifted kids, but it’s going to be measured most reliably by what we do for poor kids, kids that are most vulnerable, kids that are abused, kids that are coming out of these facilities.

These are the children that have the greatest impact on the overall health of the community because in many ways they sometimes hold some of the most substantial and significant problems. So, you can’t really claim to want to improve the health of the community if you’re only focusing on the people who are more privileged and sometimes arguably less vulnerable.

But even beyond that, the second thing I would say is the complex problems, the serious problems that we see in the lives of children that are in these settings, understanding that, evaluating that, treating that, curing those problems is the way in which we increase our capacity to cure and provide healing and treatment for everybody.

When we can help somebody dealing with severe trauma and abuse, when we can help somebody dealing with the complex challenges that many of these young people are dealing with, we learn things about human behavior, we learn about the efficacy of certain kinds of interventions and drugs, we learn about behavioral strategies that allow us to then be better practitioners for everybody. However, the opposite doesn’t work, failing this population undermines care for everyone.

When you’re only dealing with a population of children who don’t have trauma, don’t have abuse, don’t have these kinds of complex issues you’re not going to learn anything about what you need to learn to deal with these more complex problems.

And so, from just a health efficiency, a health quality perspective, you’re going to do more by working with this population to learn what works, what’s effective, what’s a good intervention, what’s a bad intervention than you will without this population.

And then I’d say the last thing is that when you help children who are the most at risk, the most vulnerable, the most in need, I do think you help everybody. I think this is an intervention that’s around health, but it ultimately creates public safety because these kids are going to interact better with other kids who have less health challenges but are equally vulnerable.

And it’s like, you know, you think about infectious disease. You know, if you’re trying to stop the spread of COVID, but you only want to work with people who don’t have COVID, and you don’t want to see anybody that has it, you’re not going to be effective at containing that contagion.

And many of these children are dealing with chronic and serious problems that if we don’t learn to be responsive to, if we’re not responsive to, the whole health of the entire community is implicated. And so, I’ve always seen health as the most effective approach to public safety that we can identify. And that’s true for adults. It’s particularly true for children.

**Michael DeBaun MD, MPH:** Fantastic. I couldn’t agree with you more. A critical aspect of being a complete pediatrician is the ability to provide high quality medical care to the most vulnerable underserved child. This skill is tantamount to being a complete pediatrician.

**Bryan Stevenson JD:** Yes

**Michael DeBaun MD, MPH:** So, the next question is a loaded question. I have a premise that our generation of pediatricians has done well in trying to improve the lives of children. However, we have not paid attention to this population of children and adolescents held in custody. I believe the best efforts in this area of health care have yet to come. The current cohort of medical students, law students, and recent graduates across the United States, will push the agenda that we have a moral contract to treat the least fortunate of our children. Do you agree?

**Bryan Stevenson JD:** Yes.

**Michael DeBaun MD, MPH:** How do we engage this young, motivated, fired up population of medical and law students, so that they cannot be siloed like you and I were when we went through graduate school, but recognize that the true strength is in the multi-disciplinary approach to engaging this vulnerable high-risk population. What would be the approach that you believe we could entertain to support a medical student and law student starting their journey in their respective professional school, August 1st of 2024?

**Bryan Stevenson JD:** Yeah, that’s a great question. It’s a great question. I think the first thing is to affirm for young professionals that this is something they can do and that it is as legitimate, credible, reputable, and even honorable as all the other options that they’re offered.

I think medical schools and law schools have not done well in creating paths to service, paths to working with this population. I mean at least in law school, it’s set up. I mean they’ve accommodated every major law firm to come and interview you and there’s a whole fee and financial incentive to work with these firms. There’s a culture around hiring for major law firms. There’s a culture around hiring for judges with elite clerkships and the schools support that. They accommodate the needs of those institutions. They adjust the academic calendar to facilitate a lot of these things.

There’s just buy-in that that’s a path that students will want, and we have to support that. We haven’t done the same kind of culture to support students who want to serve, who want to work with the poor and oppressed, and I think that institutions can do better. But what I would say to young professionals is you can do it whether your institution facilitates the assistance that would make things easier or not. It’s important for you to know you can do it. And there are a lot of us out here who can affirm that for them. That’s going to be number one.

Number two is that a lot of the skills you need, a lot of the habits you need, a lot of the perspective that you need is not something that is taught in our medical and law schools. That is, you are typically taught how to be a great associate at a law firm when you graduate from most of our law schools. You’re taught how to be a great resident in a healthcare setting when you graduate from medical school.

But you’re not taught how to work with vulnerable populations that have been disserved by the healthcare industry. You’re not taught how to manage distress. You’re not taught how to deal with systemic poverty, bigotry or racism in communities that feel very oppressed by these things.

We must facilitate the kind of necessary training, education, and acculturation for our young professionals. And, you know, one of the things that’s helped in law was all of these fellowships that emerged where people could be funded to work with nonprofits and the kind of institutions that are providing services to these populations.

When I came out, you know, I had a fellowship to work with a nonprofit that my classmates organized by donating 1% of their law firm salary to this pile of money that I could access to pay me because the nonprofit I worked with didn’t have any money. And it wasn’t a lot of money, but it was basically a way for a lot of people to contribute to this effort. I’d love to see more of that developed within medicine and healthcare spaces.

I think that the fourth thing is that we just must position people in the places where they have the experience of being rewarded by the service that they provide. For me, it was going to death row without a lot of information and knowledge but being able to see the impact of just being present in someone’s life that changed everything.

The opportunity to have your life transformed by what you can do serving a population like the population we’re describing is everywhere. I mean, there’s no limit, there’s no shortage of need, and it’s connecting our young people and our talented.

And I will just say, you know, this generation of young doctors and young lawyers have skills that are off the charts in terms of their ability. They’re so much more prepared in a lot of ways than generations in the past. I think facilitating placement and helping them with these other things is often all that’s required to turn them into someone who becomes exactly the kind of practitioner that you’re describing.

**Michael DeBaun MD, MPH:** Yes, we’re in complete agreement, and the push for change may have to be from the students up, as opposed to from the administrators down approach.

**Bryan Stevenson JD:** Absolutely, I totally agree with that. When I went to Harvard Law School starting in 1981, there was no clinical legal education anywhere. The opportunity to do clinical work was very limited. It was an amazing professor there who did facilitate some of us to work at legal services near Boston. Today, almost all of the leading law schools have strong clinical legal programs. And they do it because it started mattering to students when they apply.

There were a lot of us saying, look, go some place where you get to meet clients where you actually get to work with people who have needs while you’re in law school, because that wasn’t true for my generation of law students.

And you’ve seen all of these schools now invest more and more into clinical legal education, because it’s really great, and it makes for better lawyers. But secondly, students really consider that as a priority. And they’re not going to go someplace where that is not an option. I think comparable change is something that we should be encouraging in medical school education as well.

**Michael DeBaun MD, MPH:** So, this is the last question. Our medical legal system has not acknowledged the maternal fetal dyad as a unit. So, the mother gets the prenatal care when she’s in custody. The pediatrician does not visit the mother while she’s pregnant in custody. And then you have a baby, born while in custody.

What do you see as a viable solution that would allow us to acknowledge the importance of the dyad as a single healthcare unit, as opposed to the pregnant women being in one silo, the fetus being in another silo, and then the baby born being in a third silo?

**Bryan Stevenson JD:** Well, I think it’s a fantastic question, and I like your frame that we have to see this as a whole dyad with multiple components. I think we must fundamentally change the narrative about maternal health care, health care for pregnant women, pregnant people, and babies.

I just think all of that must shift in a radical way. And I don’t think it’s crazy to say, when we’re dealing with somebody who is pregnant, they’ve got unique health needs that we have to prioritize.

We can’t say, oh no, you shoplifted, or you used drugs, or you did something. We’ve been prioritizing punishment crime even for people with critical health needs. I think we have to prioritize their health situation and provide a setting that allows them to achieve the bonding that you’re talking about; to get the care they need during pregnancy, and then to provide for the health of the baby.

It’s about shifting priorities. We have radically shifted policy in the United States around birth and birthing in many places. I mean, that’s a big issue in many states but where we haven’t prioritized that is in these custodial spaces.

There are a lot of places in the world where a person who is pregnant, who is facing punishment has their punishment pushed to the side and their pregnancy, their health needs prioritized.

We do this in other contexts, but we don’t do it for pregnancy, we don’t do it for childbirth, and we don’t do it for newborns. And I just think that has to change. And, you know, what pediatricians could help others understand is if, you know, somebody shoplifted, if somebody wrote a bad check, if somebody did whatever it is that put them in custody, even somebody committed a violent crime. If we prioritize, health care for this person, and they give birth, we know that for a period of time, that parenting and that caregiving is going to dominate their life. And we can facilitate that, and we can evaluate that, and then we could assess whether there’s some debt owed to the society in a punitive way.

Very few institutions are thinking about this by prioritizing health, healthy pregnancy, healthy childbirth, and healthy babies. And I think if we did that and we just let our knowledge of that shape our policies, we’d see a very different paradigm emerge and much healthier people.

And the other part that I’ll add, it would also make caregiving and institutional oversight by staff healthier as well. Because if you work in these facilities and you have to separate a child from the birth parent, if you have to facilitate all of these things that don’t allow that bonding, knowing that that bonding is important, it’s hard to feel great about the job you have. You can’t feel great about the work you do. It actually deteriorates the health of everybody involved to be complicit in something that is so destructive of a healthy future. And that’s why I think we have to shift this paradigm entirely.

**Michael DeBaun MD, MPH:** And I would say the action would be cruel and an unusual punishment to the newborn to separate the newborn from their mother. The American Academy of Pediatrics, the American Academy of Family Physicians, the World Health Organization, and UNICEF all advocate for skin-to-skin care and continuous, unrestricted contact between medically stable mothers and their newborns immediately after birth.

**Bryan Stevenson JD:** Yes, absolutely.

**Michael DeBaun MD, MPH:** The newborn is looking for the breast, the newborn is looking for the mother’s warmth, to be laid on the mother’s chest, just to go to sleep, just to be coddled.

**Bryan Stevenson JD:** That’s right.

**Michael DeBaun MD, MPH:** And the absence of the maternal-newborn bonding period is cruel to that infant. There’s no other way to describe it. The newborn has a human need. And to deny that newborn this basic need doesn’t reflect the best in us.

**Bryan Stevenson JD:** That’s right. Totally agree. Totally agree.

**Michael DeBaun MD, MPH:** So, do you have anything that you would like to ask me, any pending questions?

**Bryan Stevenson JD:** I just want to express gratitude to you and others that have elevated the needs of children in custodial settings, mothers and parents in these settings.

It’s long overdue, and I just think there’s a lot of good we can do for the health of our communities, for the safety of our communities, and for the health of our children and their parents by a more intentional commitment to this area.

So, I just want to express my gratitude to you for your leadership in this area and how excited I am to see what can come from this in the years to come.

**Michael DeBaun MD, MPH:** Thank you for the partnership. I know when I came to Mongomery, Alabama, to meet you at the Equal Justic Initiative office two summers ago, I’m sure you were like, who is this guy? He seems like a medical Don Quixote. He wants to do something that hasn’t quite been a strong component of the medical culture. And he wants me to show up in Toronto for an hour lecture and meet with people an hour before the invited lecture, and work on the APS issue of the year in 2023-24, Improving the Quality of Health Care for Children and Adolescents in Custody.

So, I appreciate the trust and really the faith that our effort is bigger than me. And I just happen to be at the podium as the APS president to echo the needs of our children who we care so much about. Thank you, we all look forward to working with the Equal Justice Initiative going forward.

**Bryan Stevenson JD:** Wonderful, wonderful. Thank you, my friend.
